# Targeted radionuclide therapy with astatine-211: Oxidative dehalogenation of astatobenzoate conjugates

**DOI:** 10.1038/s41598-017-02614-2

**Published:** 2017-05-31

**Authors:** David Teze, Dumitru-Claudiu Sergentu, Valentina Kalichuk, Jacques Barbet, David Deniaud, Nicolas Galland, Rémi Maurice, Gilles Montavon

**Affiliations:** 1 SUBATECH, UMR CNRS 6457, IN2P3/IMT Atlantique/Université de Nantes, 4 rue Alfred Kastler, BP 20722, 44307 Nantes Cedex 3, France; 2 CEISAM, UMR CNRS 6230, Université de Nantes, 2 rue de la Houssinière, BP 92208, 44322 Nantes Cedex 3, France; 3 Centre de Recherche en Cancérologie de Nantes-Angers, Inserm U892, CNRS UMR 6399, Université de Nantes, Institut de Recherche en Santé de l’Université de Nantes, 8 quai Moncousu, F-44007 Nantes Cedex 1, France; 4 GIP ARRONAX, 1 rue Aronnax, F-44817 Saint-Herblain, France

## Abstract

^211^At is a most promising radionuclide for targeted alpha therapy. However, its limited availability and poorly known basic chemistry hamper its use. Based on the analogy with iodine, labelling is performed via astatobenzoate conjugates, but *in vivo* deastatination occurs, particularly when the conjugates are internalized in cells. Actually, the chemical or biological mechanism responsible for deastatination is unknown. In this work, we show that the C−At “organometalloid” bond can be cleaved by oxidative dehalogenation induced by oxidants such as permanganates, peroxides or hydroxyl radicals. Quantum mechanical calculations demonstrate that astatobenzoates are more sensitive to oxidation than iodobenzoates, and the oxidative deastatination rate is estimated to be about 6 × 10^6^ faster at 37 °C than the oxidative deiodination one. Therefore, we attribute the “internal” deastatination mechanism to oxidative dehalogenation in biological compartments, in particular lysosomes.

## Introduction

Halogens are usually named according to ancient Greek words denoting one of their characteristics. The heaviest of these elements befell the name astatine, as a reference to its instability^1^. While this name was proposed from a physical perspective (*i*.*e*. the absence of any stable isotope), one might tend to consider that it is also suitable from a chemical point of view, since most bonds involving this atom are less stable than the ones involving its closest analogue, iodine. This affects the potential application of ^211^At to medicine. Nevertheless, ^211^At is considered as one of the most promising radionuclides for targeted alpha therapy, due to its favourable physical properties (notably its half-life time of 7.2 h and its α-particle emission yield of 100%)^2, 3^.

Clinical trials using either monoclonal antibodies (mAbs) or antibody fragments labelled by astatobenzoate conjugates afforded encouraging results against both recurrent brain tumours and recurrent ovarian cancers^2, 4–6^. However, labelling with astatobenzoate conjugates suffers from *in vivo* dehalogenation, which diminishes the tumour uptake and leads to the release of free astatine and its accumulation in stomach and thyroid. Even if stomach and thyroid uptake can be mitigated^7–9^, a more stable labelling is needed for systemic administration^8^. It is particularly interesting to note that when mAbs are labelled with astatobenzoates, deastatination is limited^10–14^, while when antibody fragments are used, considerable dehalogenation occurs^10, 11, 15^. Unfortunately, the slow mAbs pharmacokinetics are not well-suited to be combined with the ^211^At half-life time^7, 16^, and astatine-labelled antibodies have been so far limited to locoregional treatments. Moreover, this behaviour is remarkable, as it echoes the one of proteins, iodinated through direct labelling or using the Bolton-Hunter reagent^17^. In these cases the dehalogenation mechanism has been elucidated: the *2*-iodophenol moiety of the catabolites released through carrier metabolization undergoes dehalogenation catalysed by deiodinases. Thus, the deastatination mechanism is most probably initiated through internalization into cells and lysosomal degradation. To overcome deiodination, reagents such as the *N*-succinimidyliodobenzoate (SIB) have been synthesized by electrophilic radioiodination of *N*-succinimidylaryltrialkylstannane derivatives^18, 19^. Indeed, these reagents lack the phenolic hydroxyl group required in the catalytic mechanism of mammal deiodinases^18–21^.

Therefore, an analogous astatination reagent, the *N*-succinimidylastatobenzoate (SAB), has been developed^10^. However, the aforementioned studies of astatobenzoate-labelled proteins showed that such labelling with ^211^At is unstable, leading to dehalogenation, contrary to the iodine case. Hence, it is clear that carrier catabolism favours astatobenzoate deastatination via mechanisms that remain unknown. It should also be mentioned that besides proteins, the injection of small organic compounds such as astatobenzoate-labelled biotin derivatives^22^ or simply 3-astatobenzoate itself^11^ leads to a radioactivity biodistribution similar to that of astatide, and very dissimilar to the iodobenzoate one, denoting a fast deastatination. Alternatively, labelling with astatodecaborates instead of astatobenzoate conjugates was envisaged, leading to stable labelling even with small molecules and very encouraging preclinical results^23–25^. However, this approach seems hindered by high uptake in kidneys and liver^7, 26^. Note that the study of such compounds is beyond the scope of the present work, which aims at revealing the mechanism(s) responsible for astatobenzoate dehalogenation.

Two explanations have been proposed so far to justify the astatobenzoate dehalogenation, (i) the action of unidentified enzymes that would catalyse the C−At bond cleavage (similarly to what happens to radioiodinated proteins by direct labelling)^27^, and (ii) the relative weakness of the C−At bonds compared to the C−I ones. One may argue that since astatine is absent from the biosphere (it is the rarest naturally occurring element on Earth)^28, 29^, no At-specific enzyme that catalyses C−At bond breakages is likely to exist. However, some proteins such as the sodium-iodide symporter recognize both astatide and iodide^30, 31^, demonstrating that the presence of iodine-processing enzymes may also affect astatine compounds. Nevertheless, C−At bond cleavages induced by promiscuous enzymes are unlikely to happen since the analogous C−I bonds are not cleaved. The second and most often quoted justification, that the C−At bonds are weaker than the corresponding C−I ones, although true^32^, is not sufficient to explain why astatobenzoate-labelled proteins are seemingly stable in blood and not when internalized inside living cells.

In order to explain the biodistribution observations from the literature, the sought *in vivo* deastatination mechanism(s) should satisfy the following criteria, (i) the analogous C−I bonds must remain stable under conditions that are sufficient for allowing C−At bond cleavages, (ii) these conditions could not be met in blood, but rather in other biological compartments such as the ones the carrier and its catabolites enter during the catabolism process, and (iii) the C−At bond breakage, should occur in the absence of any enzyme. This work aims at providing a satisfactory explanation that fully meets these criteria. One of the most striking differences between I and At lies in the astatine metalloid properties^33, 34^: its Pourbaix (*E*−pH) diagram displays cationic species^35, 36^, contrarily to the iodine one^37^, and the ionization potential of its free atom^28^ is lower than the one of iodine by more than 1 eV. Therefore, one may hypothesize that astatinated compounds are more sensitive to oxidation than iodinated ones, which seems particularly relevant since carrier catabolism (which favours deastatination) exposes astatobenzoate moieties to critical changes in redox conditions. Indeed, internalization in cells will lead labelled carriers into lysosomes, where myriads of strong oxidants such as the so-called reactive oxygen species (ROS), a family of compounds including peroxides and oxygen radicals, are present.

Here, we report the stability of an astatobenzoate conjugate in the presence of various oxidants to assess if its oxidation is possible, and if it eventually leads to deastatination. Indeed, an extensive metabolic study has been made on a ^125^I-iodobenzoate-labelled antibody fragment^38^, showing the presence of iodobenzoate and of its lysine and glycine conjugates as main catabolites, free iodine being absent. One could thus assume that the astatobenzoate catabolites would be similar to the iodinated ones. Oxidants such as permanganates, peroxides or hydroxyl radicals have been tested. Also, we show that the Fenton reaction, which happens *in vivo* in lysosomes, leads to deastatination within seconds. Since astatine is only produced in minute quantities, no spectroscopic tool can be used to probe the nature of its chemical species. Therefore, we also present relativistic density functional theory (DFT) calculations to elucidate or at least get insight on the “microscopic” mechanism that eventually leads to deastatination. It is indeed of great interest to combine quantum calculations and experiments to obtain information on astatine species at the molecular scale^35, 39–41^. Finally, we conclude by attributing the deastatination mechanism to oxidative dehalogenation.

## Results

### Oxidative dehalogenation of astatobenzoate conjugates

**Figure 1:**
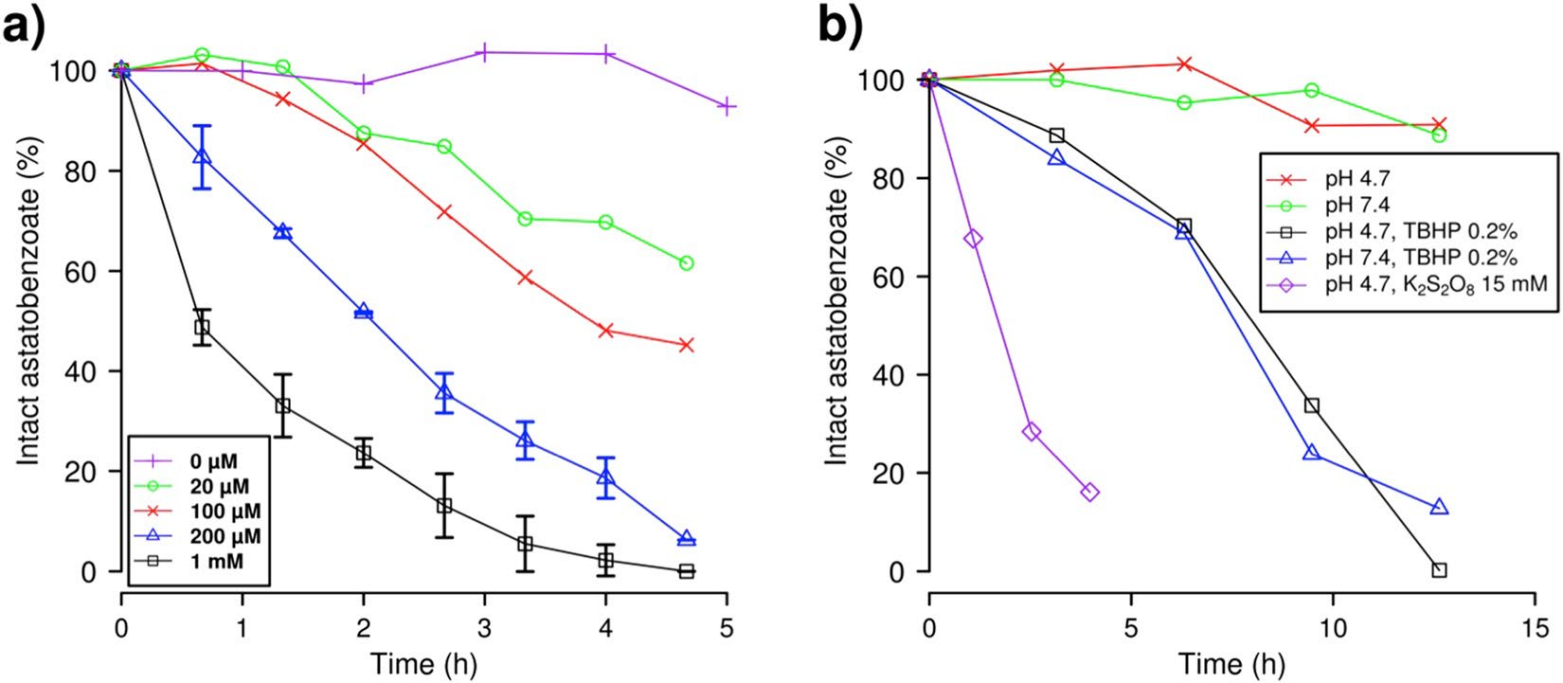
Influence of oxidants on the deastatination of ethyl 3-astatobenzoates. The proportion of intact ethyl 3-astatobenzoate is assessed by reverse-phase HPLC coupled to a dual-flow cell gamma detection system^42^. (**a**) Concentration-dependent deastatination promoted by permanganate. The NaMnO_4_ concentration is varied between 0 and 1 mM while the pH value is fixed at 4.7 with a phosphate-acetate buffer (50 mM). (**b**) Effect of peroxodisulfate (purple) and *tert*-butyl hydroperoxide (TBHP, black and blue) on the ethyl 3-astatobenzoate stability (see text).

To probe the oxidative dehalogenation hypothesis, we have selected ethyl 3-astatobenzoate (**1a**) as a model compound for performing stability studies in presence of oxidants such as permanganates (Fig. 1a) or peroxides (Fig. 1b) by measuring the proportion of “intact” astatobenzoate conjugate by reverse-phase high-performance liquid chromatography (HPLC). The influence of acidity (up to pH = 1) and strong reductants was also briefly investigated, but did not result in any noticeable deastatination.

Figure 1a clearly evidences that, at pH = 4.7 (an average pH value for lysosomes), the presence of ${{\rm{MnO}}}_{4}^{-}$ ions leads to the deastatination of astatobenzoates, in a concentration-dependent way. This demonstrates for the first time that astatobenzoate conjugates can be altered by oxidative dehalogenation, contrarily to what was previously thought^43^. By contrast, no discernible deiodination is observed after 12 h incubation of ethyl 3-iodobenzoate (**1b**) in the presence of 2 mM NaMnO_4_ (not shown). Figure 1b also shows that some peroxides (*tert*-butyl hydroperoxide, referred to as “TBHP” and peroxodisulfate) are also able to induce deastatination at the same pH value. This is particularly interesting since the most notorious oxidants occurring *in vivo* are the ROS, among which peroxides can be found. Also, one should note that oxidative dehalogenation induced by TBHP occurs at physiological pH, *i*.*e*. 7.4, with similar kinetics as observed at pH = 4.7.

### Oxidative dehalogenation of astatobenzoate conjugates induced by Fenton and Fenton-like conditions

**Figure 2:**
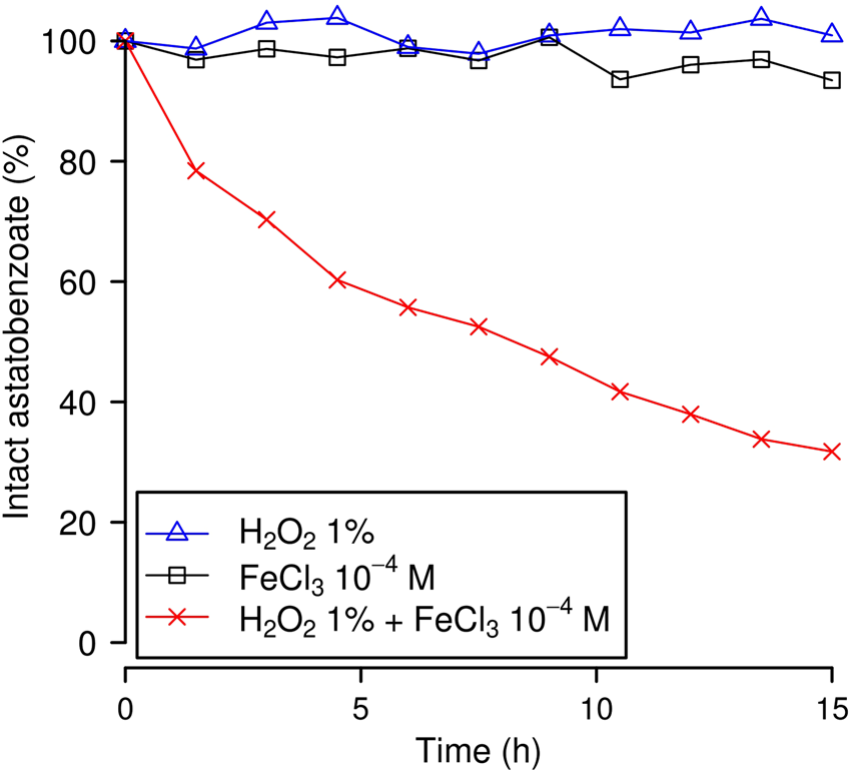
Influence of Fenton-like conditions on the deastatination of the 1a astatobenzoate. Amounts of intact ethyl 3-astatobenzoate are assessed by reverse-phase HPLC coupled to a dual-flow cell gamma detection system^42^.

The most common *in vivo* ROS, namely hydrogen peroxide (H_2_O_2_), does not promote any noticeable deastatination (see Fig. 2). By contrast, the combination of catalytic amount of ferrous iron with hydrogen peroxide (and other peroxides as well) was proven to be a powerful oxidant more than 120 years ago^44^. While radicals were not known at that time, it is now well-established that this combination produces hydroxyl radicals, which are highly reactive species^45^. When astatobenzoate conjugates are incubated under Fenton conditions (50 mM of the phosphate-acetate buffer − pH ≈ 3, 10^−4^ M of Fe^2+^ ions, and 1% H_2_O_2_), they undergo an extremely fast deastatination: within the few tens of seconds needed for the HPLC injection, most of the astatobenzoate moieties were dehalogenated, and the majority of the activity was found in a peak having a retention time that appears to match the one of an oxygen adduct of astatine (in particular an At (III) species).

Catalytic amounts of ferric iron (*i*.*e*. trivalent iron instead of divalent) coupled with hydrogen peroxide are also known to produce hydroxyl radicals, but at a much slower rate^45^. These conditions are often referred to as “Fenton-like”. The dehalogenation kinetics of ethyl 3-astatobenzoate (**1a**) incubated under Fenton-like conditions is displayed on Fig. 2. Note that, for illustration purposes, the radiochromatograms obtained after 3 h with 1% H_2_O_2_ and 1% H_2_O_2_ plus 10^−4^ M of Fe^3+^ ions are displayed in Fig. S1. These experimental results prove that astatobenzoate conjugates are sensitive to oxidation via the Fenton reaction, that is actually at play in lysosomes^46, 47^. They also indicate that the released astatine species should be an oxygen adduct, but they do not indicate how and why oxidation leads to deastatination. One should note that this part of the study has been performed at pH = 3 to favour the efficiency of the Fenton reaction in order to probe the effect of the presence of hydroxyl radicals (which are produced in lysosomes) on the dehalogenation mechanism(s). Furthermore, one should note that no discernible deiodination of ethyl 3-iodobenzoate (**1b**) is observed after 12 h incubation under Fenton-like conditions. In order to gain more insight on the oxidative dehalogenation mechanism(s) and on the differences between the C−At and C−I bonds of interest, a quantum mechanical study was performed.

### Accuracy of the computational approach

As no spectroscopic information can be obtained at ultratrace concentrations, it is necessary to consider quantum mechanical calculations to get any “microscopic” information on the deastatination mechanism(s). It is of particular importance to treat spin-orbit coupling (SOC), since this relativistic interaction has a strong influence on the geometries and properties of At compounds^40, 48^. The B3LYP hybrid exchange-correlation functional was selected, owing to its “safe choice” label for investigating astatine species^48^. Before studying the bond energies of interest, it was worth checking the validity of the used level of theory on well-known systems for which experimental data are available^49^. In the case of astatobenzene and iodobenzene, the correct dissociation limit is the homolytic one (i.e. radical fission, A − B → A^·^ + B^·^), and test calculations confirmed that this dissociation limit is found to be favoured by more than a hundred kcal.mol^−1^ compared to ionic limits at the considered level of theory.

A good agreement between the experimental dissociation energies of astatobenzene and iodobenzene (44.9 ± 5.1 and 61.1 ± 4.7 kcal.mol^−1^, respectively)^49^ and the computed ones (44.7 and 59.6 kcal.mol^−1^, respectively) was obtained. Furthermore, first ionisation potentials (*IP*_*1*_s) were computed as a model descriptor for the oxidation propensity of halobenzoates. We obtained an *IP*_*1*_ value of 195.5 kcal.mol^−1^ for iodobenzene, which fits well with the experimental value of 201.3 ± 0.7 kcal.mol^−1^ ^50^. All these results provide a firm ground to the used level of theory prior to starting the study of the oxidation of halobenzoate conjugates. Note that for the interested reader, additional calculations to illustrate the importance of SOC on the computed quantities are reported in Tables S1 and S2. Since reliable values can only be obtained when SOC is accounted for, we only report in the main text results that do include this relativistic interaction.

### Probing the sensitivity to oxidation of halobenzoates: first ionisation potentials

To probe the relative feasibility of the oxidation of **1a** and **1b**, we first computed their *IP*_1_s. At first, we checked that these *IP*_1_s were indeed related to electron removal at the halogen moiety of halobenzoates by computing the condensed-to-atom Fukui index, a quantity that resides in the realm of conceptual DFT. This index is computed through a finite difference approximation to the so-called Fukui function; for an electrophilic attack, it has the *f*_*k*_^−^ = *q*_*k*_(*N*) − *q*_*k*_(*N* − 1) form, where *k* is an atom, *q*_*k*_(*N*) is the electron population of the *k* atom in the neutral system (*N* electrons) and *q*_*k*_(*N* − 1) is the electron population of the *k* atom in the ionized system (*N* − 1 electrons). Note that the electron populations are obtained in the present work from natural population analyses. We found that the condensed-to-atom Fukui index, *f* ^−^^51^, is not only maximum for At in **1a**, and for I in **1b**, but is also at least four times larger for At or I, respectively, than for any other atom of the system. The corresponding *f* ^−^ values are 0.7 and 0.5, meaning that the removed electron is more than 50% localized on the halogen moiety in each case.

It appears that the iodobenzoate compound **1b** presents a higher *IP*_1_ (196.2 kcal.mol^−1^) than the one computed for astatobenzoate **1a** (185.8 kcal.mol^−1^). The important difference, 10.4 kcal.mol^−1^, makes **1a** actually significantly easier to oxidize than its iodine counterpart. Indeed, the previous difference is greater than the one between iodobenzene and bromobenzene, and even the one between iodobenzene and chlorobenzene (5.8 and 7.8 kcal.mol^−1^ according to the experimental *IP*_*1*_ *s*)^50^. Thus, our calculations support the fact that astatobenzoates should be more prone to oxidation than their iodinated counterparts, since the first conceptual step of any potential oxidation, *i*.*e*. the withdrawal of one electron, is more favourable in the X = At case. Therefore, since we do not aim at fully elucidating the oxidation mechanism(s), we continue by directly studying the consequences of oxidation on the C−X bonds of interest.

### The effect of oxidation on the C−X bond dissociation energies

**Figure 3:**
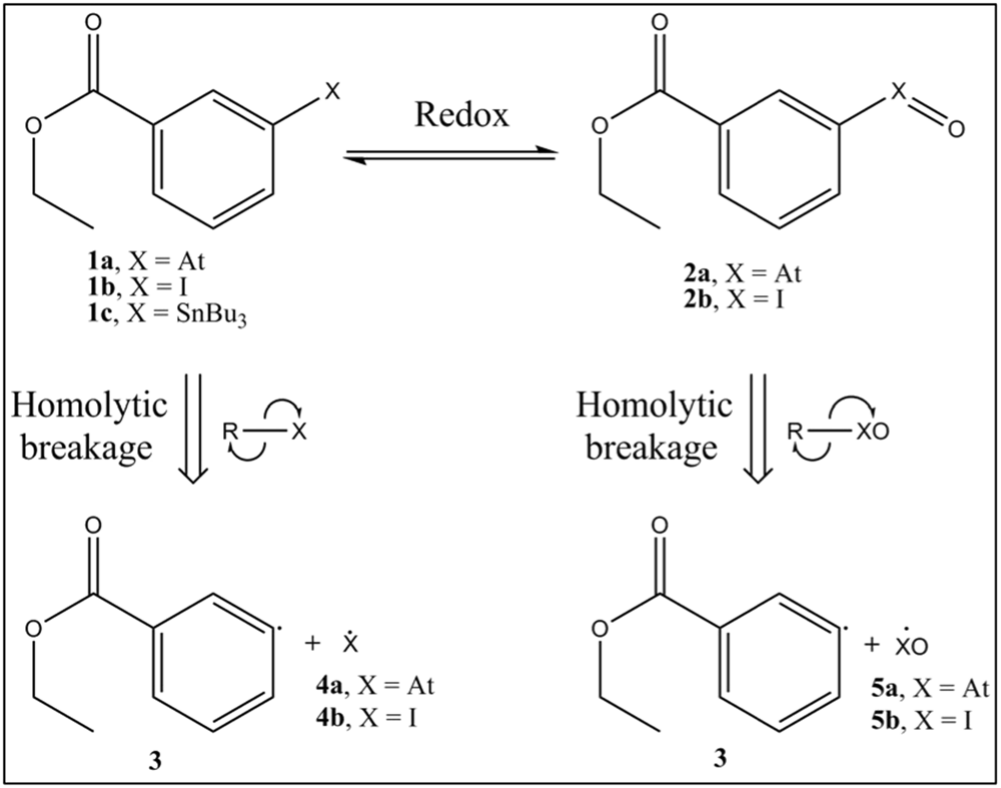
Scheme to assess the effect of oxidation on the C−X bond dissociation energies of halobenzoate compounds (X = At, I).

As the immediate product of deastatination resulting from the Fenton reaction is attributed to an oxygen adduct of astatine, and since the **2b** species is known to exist, it seems reasonable to study the dissociation energies of the **2a** and **2b** compounds (see Fig. 3), where the halogen atoms formally bear a +III oxidation state, and to compare them with the ones of **1a** and **1b**. The bond dissociation energies are calculated considering the most favourable process, *i*.*e* a homolytic cleavage, by subtracting the energies of the two radical products (see Fig. 3), from the energy of the whole molecule. The obtained numerical results are displayed in Table 1.

**Table 1:** C−X bond dissociation energies (kcal.mol^−1^) of halobenzoates (X = At, I).

Compound	DFT
**1a**	44.6
**2a**	28.2
**1b**	59.4
**2b**	37.8

By comparing the results obtained for **1a** and **1b** with the ones of the corresponding halobenzenes (44.7 and 59.6 kcal.mol^−1^, respectively), one can first observe that the C−At and C−I bond energies are almost unchanged. Much more enthralling is the comparison between halobenzoates and their oxidized counterparts, as the bond dissociation energies drop in **2a** and **2b** by more than a third compared to **1a** and **1b**, respectively, *e*.*g*. diminishing the C−At bond energy from 44.6 to 28.2 kcal.mol^−1^. While these energy decreases are of course noticeable, their significance may be further assessed with transition state theory in these undoubtedly kinetically controlled systems. As test calculations showed no energy barrier on the potential energy surfaces corresponding to the homolytic dissociations of the C−X bonds in **2a** and **2b**, as well as in **1a** and **1b**, these bond breakages are kinetically controlled by the corresponding bond dissociation energies. According to the Eyring equation^52^, the ratio between the bond breakages rates in **2a** and **2b** is affected by the following temperature-dependent factor:

1 $$k(T)=\,{e}^{\frac{{{\rm{E}}}_{{\rm{d}}}(2{\bf{b}})-{{\rm{E}}}_{{\rm{d}}}(2{\bf{a}})}{RT}}$$

where E_d_(**2b**) and E_d_(**2a**) are the bond dissociation energies of **2b** and **2a**, respectively, *R* is the universal gas constant and *T* is the temperature. Hence, the 9.6 kcal.mol^−1^ difference between E_d_(**2b**) and E_d_(**2a**) leads to a dissociation rate in **2a** larger by a factor of roughly 6 × 10^6^ at 37 °C (human body temperature) than in **2b**, which could explain the iodobenzoate relative stabilities towards oxidizing conditions compared to the astatobenzoate ones. Indeed, the iodosobenzoates will not only be produced much less efficiently than their astatinated counterparts, but also the astatosobenzoates are more prone to homolytic dehalogenation, unlike the iodosobenzoates for which the halogen will be more efficiently reduced back while proteins are oxidized^53^. On the other hand, the 16.4 kcal.mol^−1^ difference between E_d_(**1a**) and E_d_(**2a**) leads at 37 °C to an impressive relative increase of the dissociation rate for **2a**, by a factor of roughly 4 × 10^11^ with respect to **1a**. This difference is compatible with stable astatobenzoates in blood, given its antioxidant protections^54^, and efficient dehalogenation of astatosobenzoates after the oxidation of astatine to its +II oxidation state.

## Discussion

We report the first experiments of astatobenzoate dehalogenations, and shed light on the probable *in vivo* mechanism by which these therapeutically relevant compounds are catabolized. We propose that the *in vivo* C−At bond cleavage occurs through oxidative dehalogenation of the astatobenzoate moiety. This would explain the stability of the labelled carriers in blood, where the conjugates are protected from oxidative dehalogenation, notably by the thiolates of the human serum albumin (≈43 g.L^−1^) in its mercaptalbumin form, and by the strong antioxidants properties of erythrocytes^54^. Also, no strong oxidant is known to be present in blood. On the other hand, after internalisation in cells, the halobenzoates moieties would no longer be shielded from encountering strong oxidants.

We propose reactions with ROS in lysosomes as the most likely path to oxidation. Indeed, lysosomes are the organelles that are responsible for protein degradation, and will be met in the first step of the carrier catabolism. They are known to be acidic (with average pH values ranging from 4.5 to 5)^46^ and more oxidizing than other subcellular organelles^55, 56^. However, the overall oxidation level is not the only parameter at play. Indeed, strong oxidants and reductants coexist in the lysosomes microdomains^56, 57^. Similarly, the pH value experiences strong local variations, notably in the strict vicinity of proton pumps. Thus, when catabolized, the astatobenzoate conjugates will be exposed to strong oxidants, typically under acidic conditions in lysosomes. We have shown that the most common *in vivo* ROS, *i*.*e*. hydrogen peroxide (H_2_O_2_), does not intrinsically promote any noticeable deastatination. When coupled with ferrous (Fenton conditions) or even of ferric (“Fenton-like” conditions) ions, it promotes a fast cleavage of the C−At bond. It is thus clear that astatobenzoates undergo an extremely fast deastatination in the presence of hydroxyl radicals, ROS known to exist in lysosomes as products of the Fenton reaction. Indeed, due to the degradation of iron-containing macromolecules, many lysosomes are rich in redox-active iron compounds, which results in Fenton-type reactions in these organelles^46, 47^. The ubiquity of lysosomes in mammalian cells is also consistent with the observed deastatination after cell internalisation regardless of the nature of the cells.

Alternatively, another possible oxidation path for halobenzoates is worth mentioning. Indeed, P-450 cytochromes (CYPs) catalyse the oxidation of iodobenzene into iodosobenzene^53^, akin to the conversion of **1b** into **2b**. Moreover, it has also been shown, within the halobenzene series, that, the heavier the halogen, the easier it is for CYPs to oxidize it ref. 58. Therefore, it seems possible that CYPs catalyses the oxidation of astatobenzoates as well. Note that even though iodosobenzene is formed *in vivo*, it could not be abundant, as it is reduced while it oxidizes proteins^53^, and that no important dehalogenation of the iodosobenzene has been reported. It is therefore possible that following carrier metabolization, the astatobenzoates conjugates are released in the blood circulation, then captured by the liver, and later undergo oxidation catalysed by CYPs, which ultimately yields to deastatination.

The oxidative dehalogenation hypothesis nicely meets the criteria we proposed: deastatination by oxidation has been proven to be easily doable in the absence of any enzyme; it explains why the astatobenzoate conjugate stabilities must be very different in blood, where they are protected from oxidation, and within lysosomes where they are exposed to Fenton conditions (or alternatively after catabolization of the carrier and oxidation by CYPs in the liver). It is also consistent with the observed stability of the corresponding iodobenzoates under the same *in vivo* conditions: they are stable under conditions that are sufficient to provoke deastatination.

Besides experimental evidences and a proposed *in vivo* mechanism, we also aimed at giving an insight of the oxidative deastatination process at the molecular level, especially in regard to iodinated analogues of astatobenzoates. We hypothesized that the difference in *in vivo* stability between iodobenzoate- and astatobenzoate-labelled proteins with respect to dehalogenation is due to (i) the different sensitivities of the At and I atoms toward oxidation and (ii) the difference in the C−X bond strengths in the oxidized compounds. A plausible scenario is oxidative dehalogenation in which the At atom is oxidized to its +III oxidation state, which weakens enough the C−At bond and eventually leads to its breakage. Our DFT calculations show that it is much easier to start oxidizing astatobenzoates than their iodinated counterparts (by 10.4 kcal.mol^−1^ according to the calculated first ionization potentials). We also show that this oxidation results in a vast decrease of the C−X bond dissociation energies, illustrated by a drop of the C−At bond energy in the astatobenzoate from 44.6 to 28.2 kcal.mol^−1^. Finally, to link this quantity to a more *in vivo* relevant one, we provide a rough estimate of the kinetic enhancement of the homolytic cleavage rate, showing that the reaction should be accelerated by a factor of about 4 × 10^11^ (a difference greater than the one between decades and milliseconds).

Finally, we deem that our results could be of interest to the conception of innovative ^211^At-labelling agents, particularly in stimulating new ideas concerning their design and screening. Indeed, just as knowledge of the deiodinase involvement was a necessary step prior to designing SIB, our research demonstrates which types of mechanism should be inhibited for obtaining stable *in vivo* labelling with ^211^At. Also, to speed up the quest for new compounds, as the *in vivo* experiments are costly, labour intensive, and time-consuming, we suggest that prior *in silico* screenings based on relativistic DFT calculations must be undertaken given both the accuracy and the cost-effective favour of this approach.

## Methods

### Radiolabelling

The synthesis of **1a** was done following the previously described methods for the synthesis of SAB^59, 60^. Briefly, to 10 µL of acetic acid were added 25 µL of 2 mg/mL of *N*-chlorosuccinimide in methanol and 1,1 mg of ethyl 3-(tri-n-butylstannyl) benzoate (**1c**) in 25 µL of methanol in an HPLC vial. Then 50 µL of ^211^At in chloroform were added (roughly corresponding to 5–10 MBq of activity). After 20 min incubation, **1a** was purified by HPLC using a Dionex Ultimate3000 HPLC device with an Interchrom C18 column piloted by the Chromeleon 6.80 software (ThermoFisher Scientific Inc.). It was coupled with a dual-flow cell gamma detection system^42^ using a γ-ray detector (raytest GABI Star) piloted by the Gina software (raytest Isotopenmeßgeräte GmbH).

### Kinetics of deastatination

1.4 mL samples of **1a** (≃2–4 MBq of activity) were incubated in various media at 20 °C directly in the HPLC apparatus (same as described above), and 50 µL samples were injected onto column for analysis. The HPLC sequence was the following: 20 s of 100% acetonitrile (ACN) at 1.05 mL.min^−1^, a 5 s gradient decrease from 100 to 0% ACN, 65 s of 0% ACN, a 90 s gradient from 0 to 60% ACN, a 720 s gradient from 60 to 72% ACN during which the flow is increased from 1.05 mL.min^−1^ to 1.3 mL.min^−1^ during 90 s after the first 6o s of the gradient, a 60 s gradient from 72 to 100% ACN, 300 s of 100% ACN, a 60 s gradient from 100 to 0% ACN during which the flow is decreased from 1.3 mL.min^−1^ to 1.05 mL.min^−1^ and 150 s of 0% CAN for a total run duration of 24.5 min. Two subsequent injections of 50 µL of Na_2_S_2_O_3_ (50 mM) and of NaMnO_4_ (2 mM) were run in a “short run mode” between two kinetic points to wash out any potential residual activity. The short run program consisted of 1 min of 100% H_2_O, 1 min of 100% H_2_O to 100% of CH_3_CN (gradient) and 2 min of 100% CH_3_CN. The sums of the counts in the “intact” astatobenzoate peak were corrected by the intrinsic decay of ^211^At (considering its 7.21 h half-life time).

### Computational methods

All the calculations have been performed in gas phase. The two-component (2*c*) DFT methods^61^ relying on relativistic effective core potentials (namely ECP28MDF and ECP60MDF for I^62^ and At^63^, respectively) and implemented in the NWChem^64^ and Turbomole^65^ program packages were used. The hybrid B3LYP exchange-correlation functional^66^ was selected, according to results of a recent benchmark study led on At compounds^48^. For treating the 25 valence electrons on both heavy atoms, we have selected triple zeta basis sets supplemented with 2*c* extensions, referred to as aug-cc-pVTZ-PP-2*c* ^62, 63, 67^. The aug-cc-pVTZ basis sets^68, 69^ have been used for the remaining atoms (C, H and O).

## Electronic supplementary material

Supporting Information
