# Supplementary material for: Targeted radionuclide therapy with astatine-211: Oxidative dehalogenation of astatobenzoate conjugates
Source: Sci Rep. 2017 May 31;7:2579. doi: 10.1038/s41598-017-02614-2 (PMC5451414; doi:10.1038/s41598-017-02614-2)
Supplement: Supplementary file 1 — Supporting Information [file 41598_2017_2614_MOESM1_ESM.docx]

**Supporting Information for:** “Targeted radionuclide therapy with astatine-211: Oxidative dehalogenation of astatobenzoate conjugates”, by David Teze, Dumitru-Claudiu Sergentu, Valentina Kalichuk, Jacques Barbet, David Deniaud, Nicolas Galland, Rémi Maurice and Gilles Montavon.

**Table S1 | SR-DFT and 2c-DFT C−X bond dissociation energies (kcal.mol^−1^) of halobenzenes (X = At, I).**

| Compound | SR-DFT^a^ | 2c-DFT^b^ | Expt.^1^ |
| --- | --- | --- | --- |
| Astatobenzene | 60.3 | 44.7 | 44.9 ± 5.1 |
| Iodobenzene | 67.4 | 59.6 | 61.1 ± 4.7 |

^a^ Calculations performed with Gaussian09.^2^

^b^ Reported from Table 1 for convenience.

**Table S2 | SR-DFT and 2c-DFT first ionisation potentials (kcal.mol^−1^) of halobenzoates and of iodobenzene.**

| Compound | SR-DFT^a^ | 2c-DFT |
| --- | --- | --- |
| Astatobenzoate **1a** | 208.3 | 185.8 |
| Iodobenzoate **1b** | 197.3 | 196.2 |
| Iodobenzene | 196.5 | 195.5^b^ |

^a^ Calculations performed with Gaussian09.^2^

^b^ Reported from the main text for convenience.

Comments:

These results are reported here to illustrate the importance of spin-orbit coupling (SOC) on the computed quantities, thus justifying the use of two-component relativistic DFT (2c-DFT) calculations for obtaining accurate results in this context. It is clear from Table S1 that the inclusion of SOC has a significant effect on the computed bond dissociation energies; typically scalar relativistic DFT (SR-DFT) calculations overestimate these quantities mainly because the SOC stabilizations of the ground state of the dissociated halogen radicals are missing, which is not compensated by the error done on the bound system. When SOC is accounted for, we obtain values in close agreement with the experiment (with less than 2 kcal.mol^-1^ of difference). From Table S2, we can see that SOC hardly affects the IPs of the iodinated species, while a significant effect is found for the astatinated one. Thus, even if the SR-DFT results for the IPs of iodinated species may be seen as reliable enough, we chose to report only 2c-DFT results in the main text to obtain reliable results for all the considered species at a same level of theory for the sake of comparison.

**Figure S1 | Radiochromatograms of 1b obtained after 3 h with 1% H_2_O_2_ (top) and 1% H_2_O_2_ plus 10^−4^ M of Fe^3+^ ions (Fenton-like conditions, bottom).** These radiochromatograms were used to generate data displayed in Fig. 2.


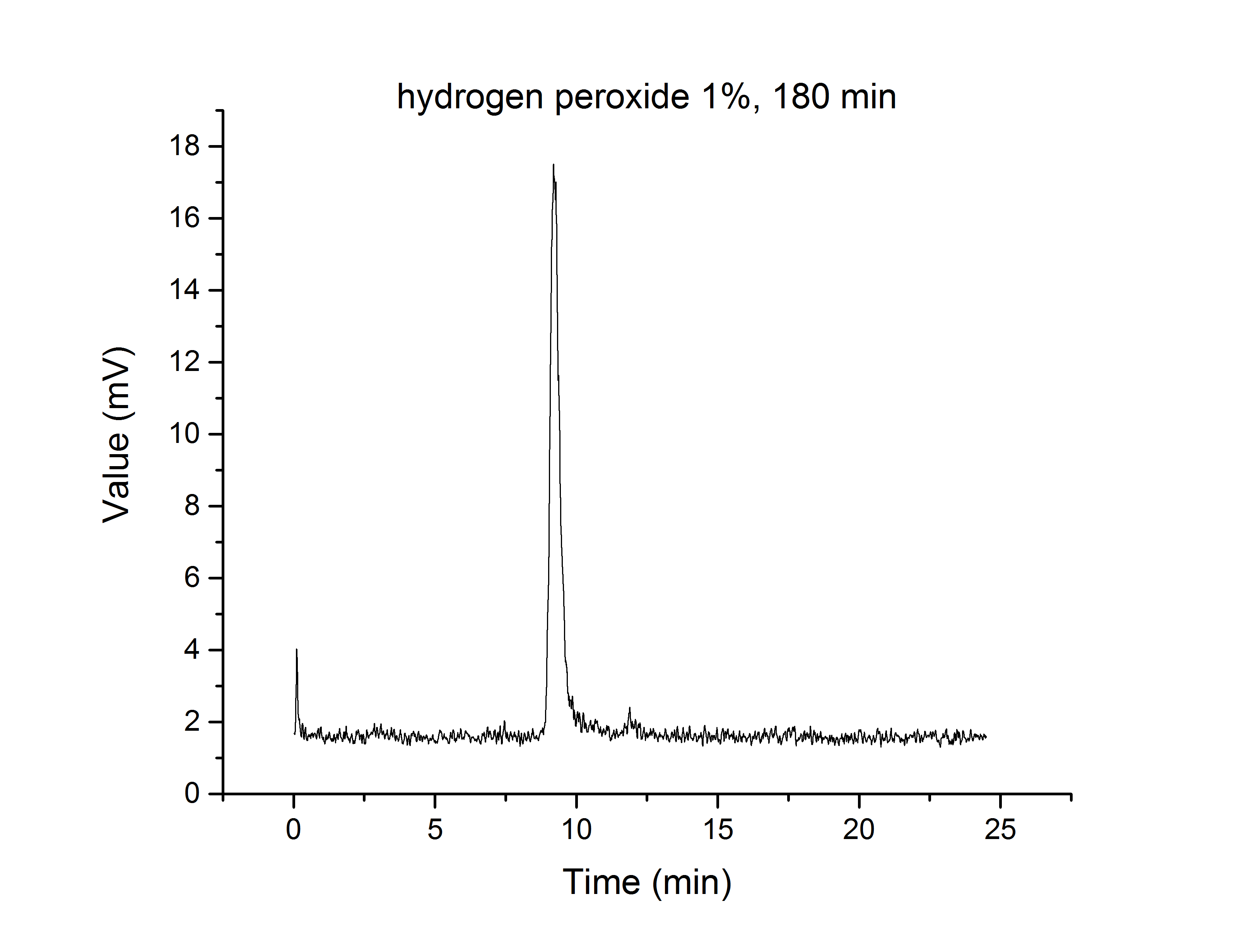


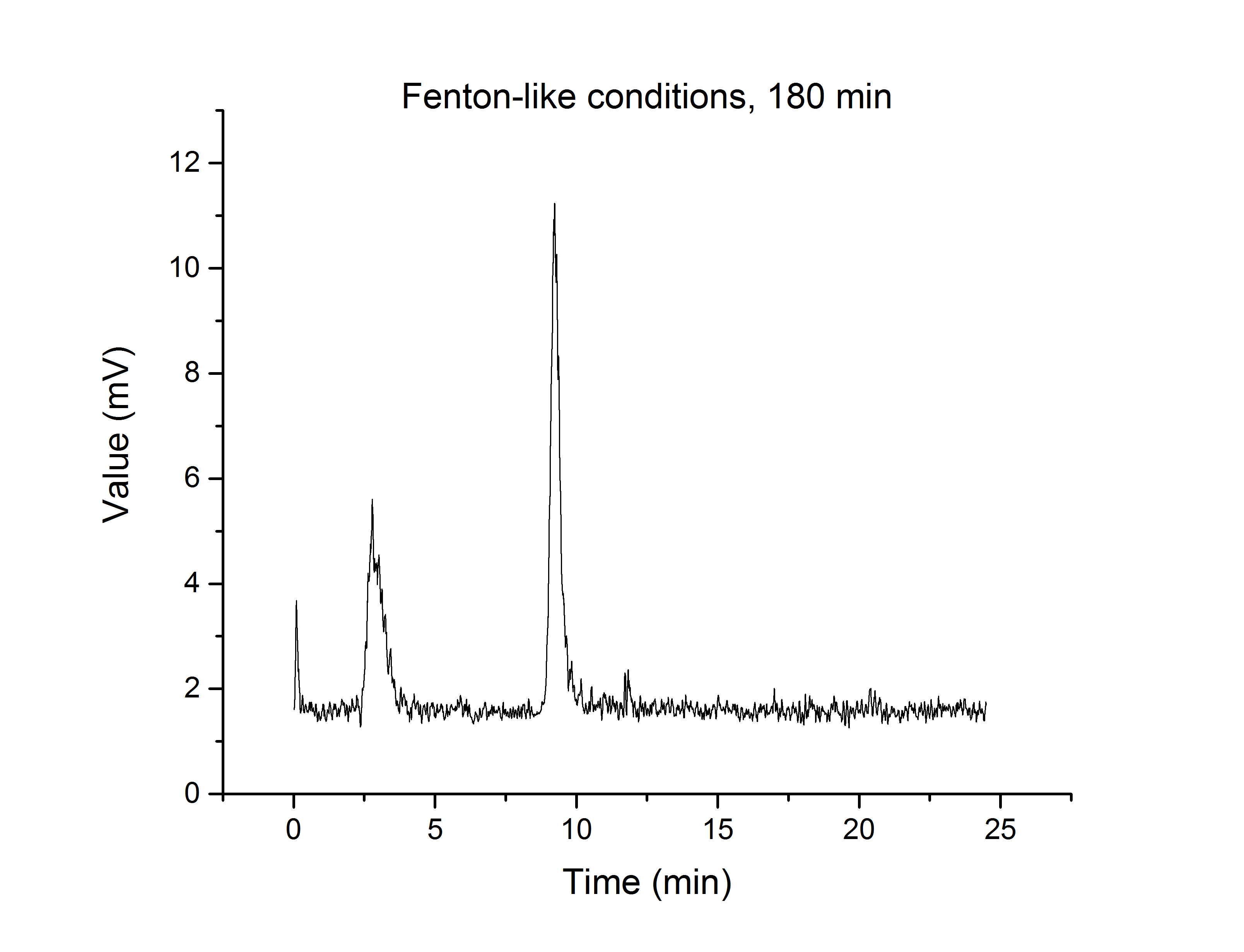


**References**

1. Вашарош, Л., Норсеев, Ю. В. & Халкин, В. А. ОПРЕДЕЛЕНИЕ ЭНЕРГИИ РАЗРЫВА ХИМИЧЕСКОЙ СВЯЗИ УГЛЕРОД-АСТАТ. *Dokl Akad Nauk SSSR* **263,** 119–123 (1981).

2. Gaussian 09, Revision D.01, M. J. Frisch, G. W. Trucks, H. B. Schlegel, G. E. Scuseria, M. A. Robb, J. R. Cheeseman, G. Scalmani, V. Barone, B. Mennucci, G. A. Petersson, H. Nakatsuji, M. Caricato, X. Li, H. P. Hratchian, A. F. Izmaylov, J. Bloino, G. Zheng, J. L. Sonnenberg, M. Hada, M. Ehara, K. Toyota, R. Fukuda, J. Hasegawa, M. Ishida, T. Nakajima, Y. Honda, O. Kitao, H. Nakai, T. Vreven, J. A. Montgomery, Jr., J. E. Peralta, F. Ogliaro, M. Bearpark, J. J. Heyd, E. Brothers, K. N. Kudin, V. N. Staroverov, R. Kobayashi, J. Normand, K. Raghavachari, A. Rendell, J. C. Burant, S. S. Iyengar, J. Tomasi, M. Cossi, N. Rega, J. M. Millam, M. Klene, J. E. Knox, J. B. Cross, V. Bakken, C. Adamo, J. Jaramillo, R. Gomperts, R. E. Stratmann, O. Yazyev, A. J. Austin, R. Cammi, C. Pomelli, J. W. Ochterski, R. L. Martin, K. Morokuma, V. G. Zakrzewski, G. A. Voth, P. Salvador, J. J. Dannenberg, S. Dapprich, A. D. Daniels, Ö. Farkas, J. B. Foresman, J. V. Ortiz, J. Cioslowski, and D. J. Fox, Gaussian, Inc., Wallingford CT, 2009.
